# Gastric Cancer Growth Modulated by circSNTB2/miR-6938-5p/G0S2 and PDCD4

**DOI:** 10.2174/1386207326666221108112113

**Published:** 2023-06-12

**Authors:** Baohai Rong, Xiqi Chen, Guangdong Xie, Letian Han, Hanhan Chen, Qingying Sun, Yongkun Zhou

**Affiliations:** 1 The First Clinical Medicine School, Shandong University of Traditional Chinese Medicine, Jinan, 250014, China;; 2 Department of General Surgery, The Affiliated Hospital of Shandong University of Traditional Chinese Medicine, Jinan, 250014, China;; 3 Reproductive and Genetic Center of Integrated Traditional and Western Medicine, The Affiliated Hospital of Shandong University of Traditional Chinese Medicine, Jinan, 250014, China;; 4 Department of Breast and Thyroid Surgery, The Affiliated Hospital of Shandong University of Traditional Chinese Medicine, Jinan, 250014, China

**Keywords:** Gastric cancer, circSNTB2, miR-6938-5p, G0S2, PDCD4, biomarker

## Abstract

**Background:**

Gastric cancer (GC) is the third most common cause of cancer-related death worldwide. Increasing studies have indicated that circular RNAs (circRNAs) play critical roles in cancer progression. However, the precise mechanism and functions of most circRNAs are still unknown in gastric cancer.

**Methods:**

In the present study, we aim to uncover the mechanism by which circRNAs regulate gastric cancer tumorigenesis. By analyzing the microarray data, we screened differential expressed circRNAs in the gastric cancer group and identified a down-regulated circRNA, hsa_circ_0040039 (circSNTB2). Mechanically, circSNTB2 served as a sponge for the miR-6938-5p and up-regulated its expression.

**Results:**

Meanwhile, G0/G1 switch gene 2 (G0S2) and programmed cell death gene 4 (PDCD4) were identified to be the aim genes of miR-6938-5p, constructing circSNTB2/miR-6938-5p/G0S2 and PDCD4 pathways.

**Conclusion:**

Taken together, our findings demonstrated that circSNTB2 plays an essential role in gastric cancer by regulating miR-6938-5p through G0S2 and PDCD4 genes. CircSNTB2 could be a promising biomarker for GC diagnosis and targeted therapy.

## 1 INTRODUCTION

Gastric cancer (GC) is one of the most common malignant tumors in the clinic and the second cause of cancer-related mortality, particularly prevalent in east Asia countries [1]. In spite of a steadily declining incidence of GC across the world, the number of new cases continues to increase in China because of population rapid growth and ageing [2, 3]. Early detection plays an essential role in GC with a significantly better prognosis compared to advanced GC [4]. However, it is difficult to diagnose because most people typically don’t show symptoms in the earlier stages and have poor awareness of risk factors [5]. Besides, less understanding of GC pathogenesis would be another obstacle to treating GC [6]. Prevention and cure of gastric cancer are still challenged in the clinic [7-9]. Therefore, searching for new molecular markers to monitor and intervene in gastric cancer carcinogenesis is urgent.

Circular RNAs (circRNAs) have been known to play an essential role not only in the ordinary development of organs and tissues [10], but also in the occurrence and progression of human diseases, including cardiovascular diseases [11], neurological dysfunction [12] and cancers [13]. Accumulating evidence has demonstrated that circRNAs are abnormally expressed in various cancer types, such as colorectal cancer [14], hepatocellular carcinoma [15] and breast cancer [16]. What is more, the role of circRNAs in the process of cancer initiation and progression has especially gained concern because circRNAs may cause cancer by interacting with tumor-associated miRNAs, proteins and genes, and by participating in pathophysiological activities. CircRNAs have been recognized as dependable diagnostic and therapeutic molecular biomarkers for cancers [17, 18]. Besides, a few researchers have focused on the correlation between circRNA and GC. Pan *et al.* have found that ciRS-7/miR-7/PTEN axis plays a significant role in gastric cancer [19]. Has_circ_0000096 and has_circ_0047905 were considered as the bioactive markers of GC due to the area under the ROC curve is 0.82 and 0.85, respectively, [20, 21]. More research should be performed to elucidate the link between circRNA and GC.

In this study, we aimed to establish the expression profile of gastric cancer through circRNA microarray chip analysis. Our data indicated that circSNTB2 is involved in gastric cancer through bioinformatics analysis. The miRNA database was further explored to identify circRNA-related dysregulated miR-6938-5p in gastric cancer. Gene ontology (GO) enrichment and Kyoto Encyclopedia of Genes and Genomes pathways (KEGG) analysis revealed the potential biology function of miRNA target genes. Finally, a circSNTB2/miR-6938-5p/ G0S2 and PDCD4 regulation network were constructed to selected hub genes and we found that circSNTB2 played a crucial role in the network.

## 2 METHODS

### 2.1 Data Collection

The circRNA and miRNA expression profiles were respectively obtained from GSE78092 (https://www.ncbi.nlm.nih.gov/geo/query/acc.cgi?acc=GSE78092) and GSE124158 (https://www.ncbi.nlm.nih.gov/geo/query/acc.cgi?acc=GSE124158). The high throughput sequencing of stomach adenocarcinoma (STAD) mRNA data was collected from Gene Expression Profiling Interactive Analysis (GEPIA, http://gepia.cancer-pku.cn/index.html). All data can be obtained online, thus the ethical statement is not required.

### 2.2 Data Processing

After batch effect normalization, the circRNA and miRNA microarray data were further analyzed by limma R package to attain differentially expressed circRNAs. P values were adjusted by false discovery rate (FDR). |log_2_(fold‐change)| >1 and FDR <0.05 were considered as significant.

### 2.3 Prediction of circRNAs Target miRNAs

Top 10 up and down-regulation circRNA-miRNA interactions were respectively predicted by circBank database (http://www.circbank.cn/index.html). Then we overlapped predicted miRNAs of up and down-regulation circRNAs, respectively. To further improve the credibility of the prediction, we intersected the predicting outcomes with the microarray data by R. And we could screen out the most potential miRNA.

### 2.4 Prediction of miRNAs Target Genes

After screening the miRNA, miRNA verified by expression profiles was considered the most likely potential target miRNA. Then, the screened miRNA was further applied to predict the target genes *via* four online databases, the Targetscan (http://www.targetscan.org/mamm_31/), miRDB (http://mirdb.org/), Encori (http://starbase.sysu.edu.cn/index.php), miRWalk (http://mirwalk.umm.uni-heidelberg.de/). Target genes of the potential miRNA from the four databases were intersected. To find the most likely potential target genes, the target genes that were predicted by the four databases simultaneously were overlapped with down-regulation genes from GEPIA.

### 2.5 GO and KEGG Pathways of Target Genes

After overlapping results from four databases, target genes were applied to Gene Ontology (GO) analysis and Kyoto Encyclopedia of Genes and Genomes pathways (KEGG) analysis through cluster Profiler R package. Ggplot2 R package was used to visualize the outcomes of the analysis. GO analysis was used for functional analysis of the genes and consisted of biological process (BP), cellular component (CC), and molecular function (MF). KEGG pathway analysis revealed signaling pathway information for the genes.

### 2.6 Survival Analysis and Expression of Target Genes

To obtain Kaplan-Meier survival curves, the most likely potential target genes were analyzed by Kaplan Meier-plotter database (http://kmplot.com/analysis/index.php?p=backgrou nd) [22, 23]. The GEPIA database is an online analytical tool based on the TCGA database. The expression of those genes was visualized by GEPIA [24].

### 2.7 Quantitative Real-Time PCR (qRT-PCR)

We used a mortar and pestle to grind the fresh tumor tissue and peritumoral tissue into fine powder under the circumstance of liquid nitrogen [25]. Total RNA was isolated by TRIzol Reagent and quantified by Nanodrop 2000 (Thermo Scientific, Rockford, IL, USA). Single-stranded cDNAs were reverse-transcribed from 1000 ng of total RNA. QRT-PCR was conducted on a CFX96 apparatus (Bio-Rad) with SYBR Green Pro Tap [26]. For each target gene, the relative expression levels of mRNAs were calculated following the 2^−ΔΔCt^ method and using 18s as a housekeeping gene (primer sequences were listed in Table **1**). Repeat at least three times for each sample.

## 3 RESULTS

### 3.1 Identification of Differentially Expressed circRNAs

**Fig. (1):**
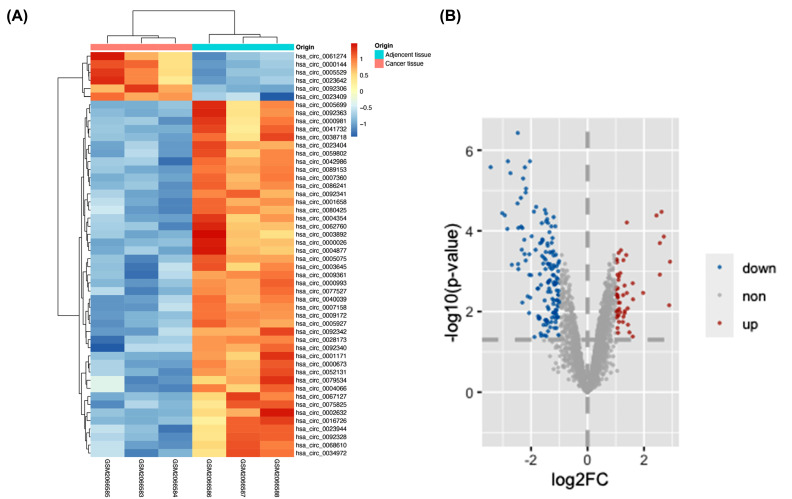
Heatmap and volcano plots for DECs based on GSE78092. **A**, Heatmap for top 50 statistically significant circRNAs with 6 upregulated circRNAs and 44 downregulated circRNAs. **B**, Volcano plot for 199 DECs. Red spots represent 53 upregulated circRNAs and blue spots represent 146 down regulated circRNAs. Black spots represent circRNAs without statistically significant change (|log2(fold change)| >1 and *P*-value <0.05).

The microarray data of gastric cancer circRNAs was from GSE78092 which used ArrayStar Human Circular RNA microarray V2.0 (GPL21485). After batch effect normalization, we used limma R package to analyze the series matrix file and discovered 199 statistically significant differentially expressed circRNAs (DECs) with 53 up-regulated and 146 down-regulated circRNAs (Fig. **1**). To enhance the accuracy of prediction, top 10 up-regulated and down-regulated circRNAs were chosen to predict target miRNAs (Tables **2** and **3**).

### 3.2 Prediction of circRNAs Target miRNAs and Expression of Target miRNA

**Fig. (2):**
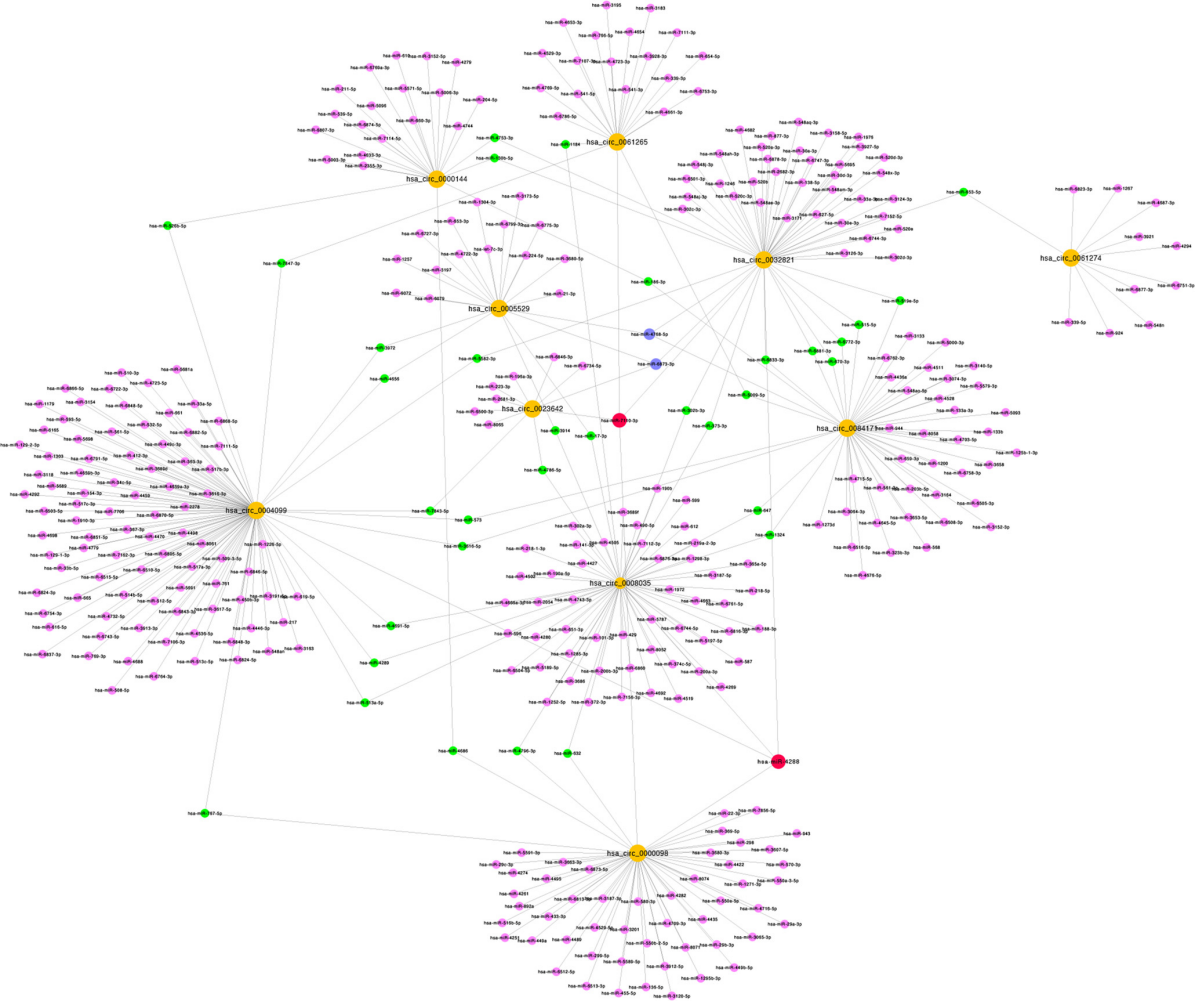
Interaction network about top 10 upregulated circRNAs with target miRNAs. Yellow spots represent circRNAs, pink spots represent miRNAs interacted with 4 circRNAs, blue spots represent miRNAs interacted with 3 circRNAs, green spots represent miRNAs interacted with 2 circRNAs and purple spots represent miRNAs interacted with 1 circRNAs.

**Fig. (3):**
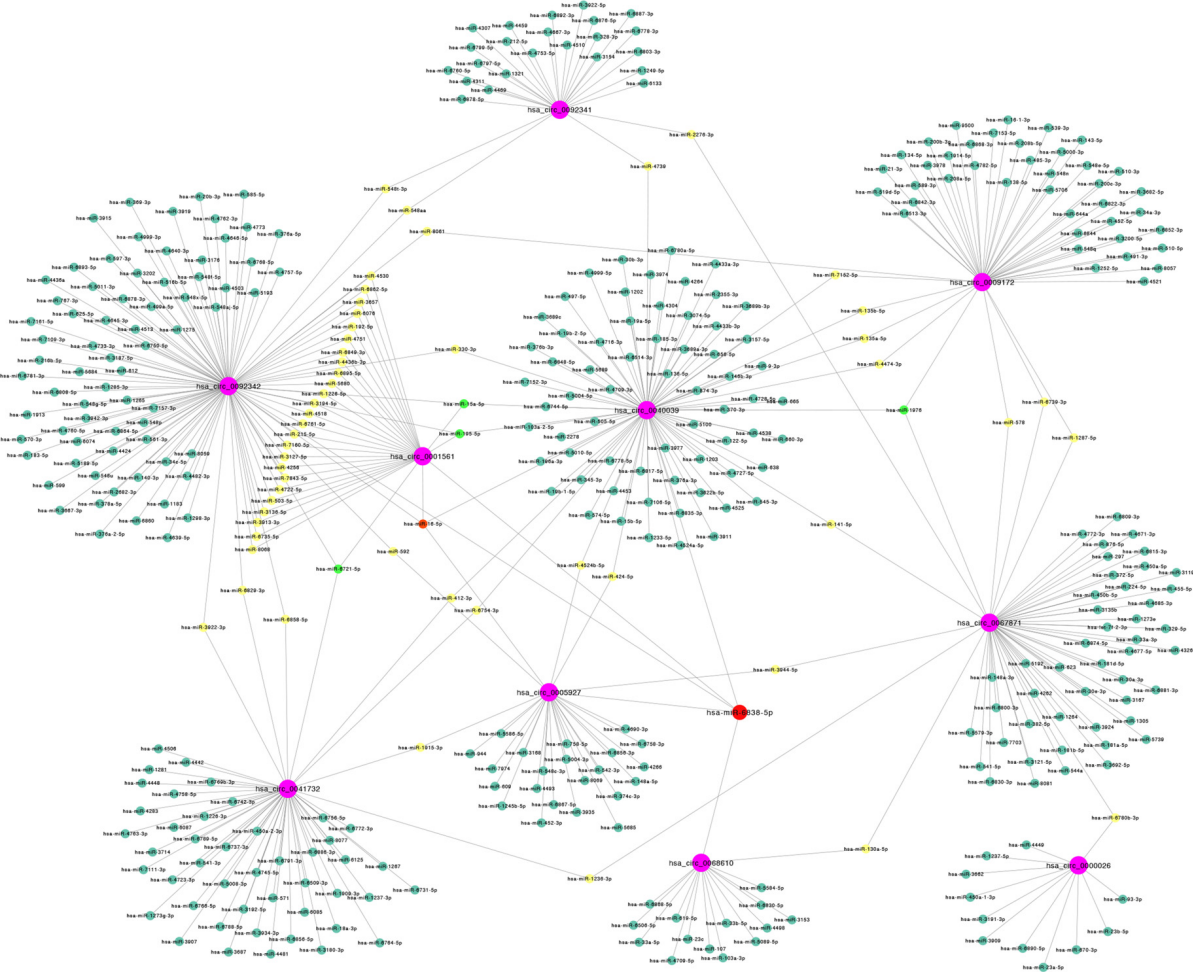
Interaction network about top 10 downregulated circRNAs with target miRNAs. Purple spots represent circRNAs, red spot represents miRNA interacted with 5 circRNAs, orange spot represents miRNA interacted with 4 circRNAs, yellow spots represent miRNAs interacted with 2 circRNAs and blue spots represent miRNAs interacted with 1 circRNAs.

**Fig. (4):**
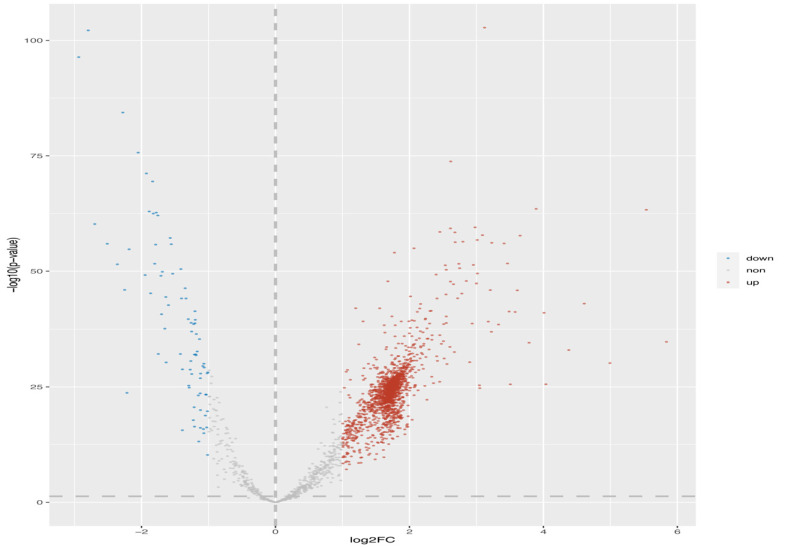
Volcano map for 1959 differentially expressed miRNAs based on GSE124158. Red spots represent 79 upregulated miRNAs and blue spots represent 1880 down-regulated miRNAs. Black spots represent miRNAs without statistically significant change.

**Fig. (5):**
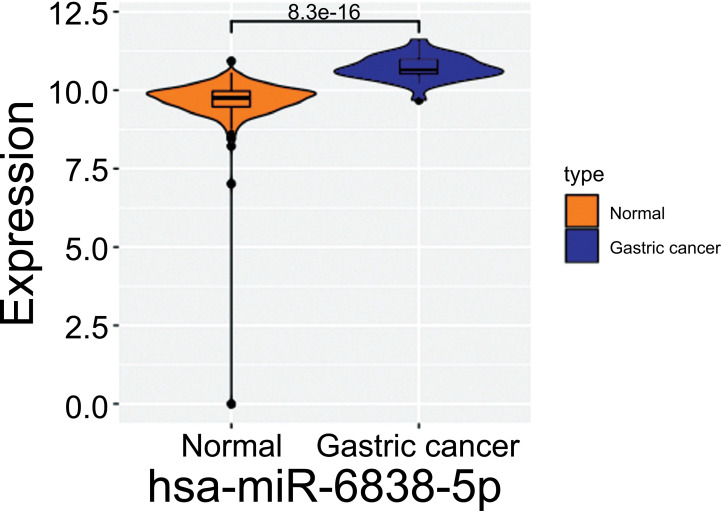
Violin plot about hsa-miR-6838-5p expression profile. Yellow module data from normal people while blue module from gastric cancer patients.

The microarray data of gastric cancer miRNAs was from GSE124158 which used 3D-Gene Human miRNA V21_1.0.0 (GPL21263). According to the predicted miRNAs from top 10 of up-regulated and down-regulated circRNAs, we found some overlapped miRNAs, which indicated that these miRNAs have more potential to be affected (Figs. **2** and **3**). Among these, hsa-miR-6838-5p has the maximum overlap numbers in the prediction of down-regulated circRNAs. Therefore, we selected the hsa-miR-6838-5p to be the target miRNA. As the most significantly down-regulated circRNAs, CircSNTB2 had the great potential to interact with hsa-miR-6838-5p. Further, we confirmed that the expression of hsa-miR-6838-5p was higher in gastric cancer group (Figs. **4** and **5**).

### 3.3 Prediction of miRNAs Target Genes and Enrichment Analysis

**Fig. (6):**
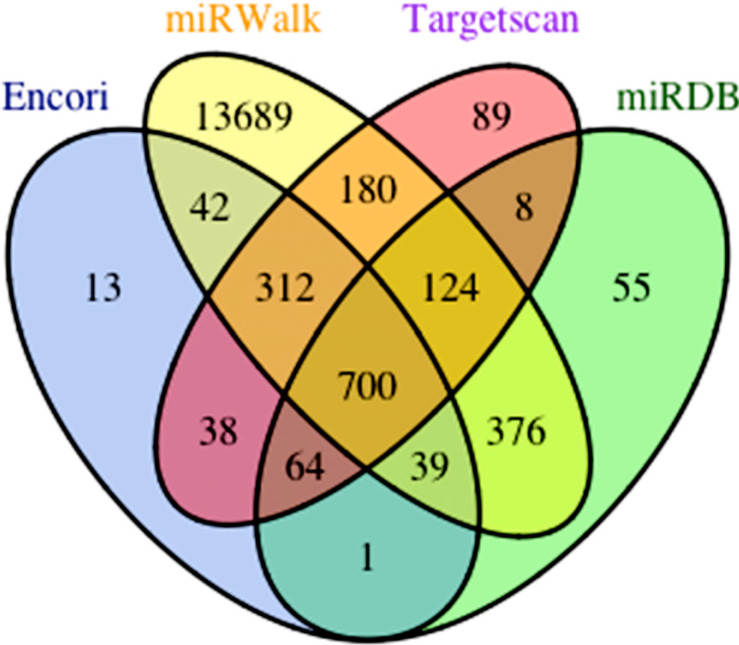
Venn diagram based on hsa-miR-6838-5p target genes from four databases (Targetscan, miRDB, miRWalk, Encori).

**Fig. (7):**
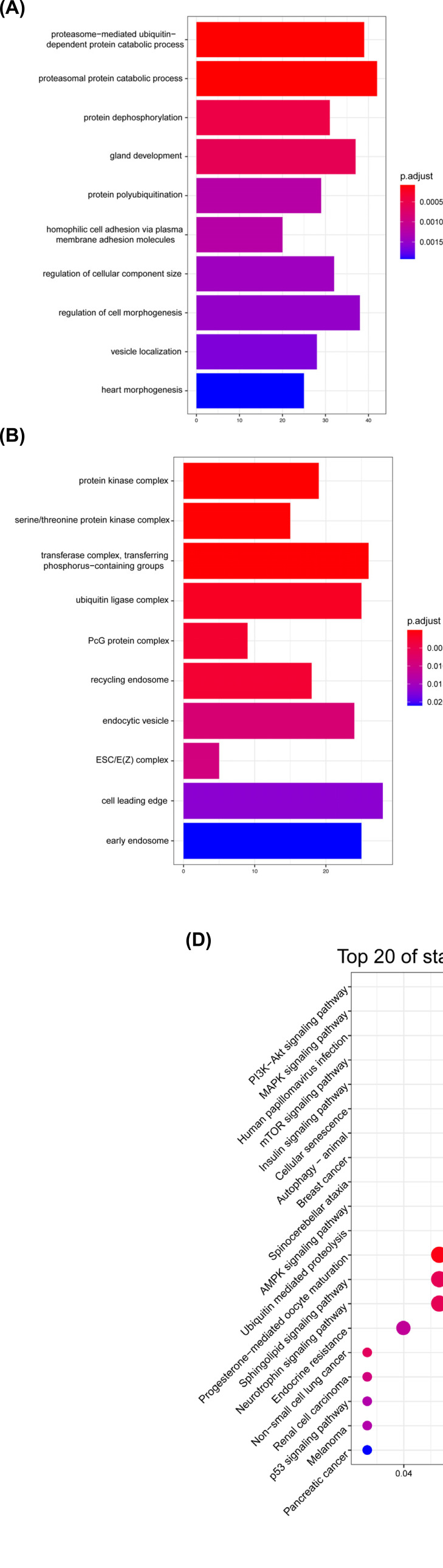
GO and KEGG analysis of 700 target genes. (**A**), BP; (**B**), CC; (**C**), MF and (**D**), KEGG pathway.

The Targetscan, miRDB, miRWalk and Encori databases were applied to predict target mRNAs. We respectively discovered 1515, 1367, 3162 and 1209 mRNAs. Further, 700 overlapped mRNAs were identified (Fig. **6**). GO and KEGG pathway analysis was completed by cluster Profiler R package and visualized by ggplot2 R package. The results of BP demonstrated that most of target genes correlated with “proteasome-mediated ubiquitin-dependent protein catabolic process”, “proteasomal protein catabolic process”, “protein dephosphorylation”, “gland development”, “protein polyubiquitination” (Fig. **7A**). Regarding CC, target genes were associated with “protein kinase complex”, “serine/threonine protein kinase complex”, “transferase complex, transferring phosphorus-containing groups”, ubiquitin ligase complex”, “PcG protein complex” (Fig. **7B**). For MF, most genes enriched in “protein serine/threonine kinase activity”, “SMAD binding”, “activin binding”, “ubiquitin-like protein transferase activity”, “ubiquitin-protein transferase activity” (Fig. **7C**). Top 5 of KEGG pathway enrichment analysis was “PI3K-Akt signaling pathway”, “MAPK signaling pathway”, “Human papillomavirus infection”, “mTOR signaling pathway”, “Insulin signaling pathway” (Fig. **7D**).

### 3.4 Screened and Quantified the Target Genes

**Fig. (8):**
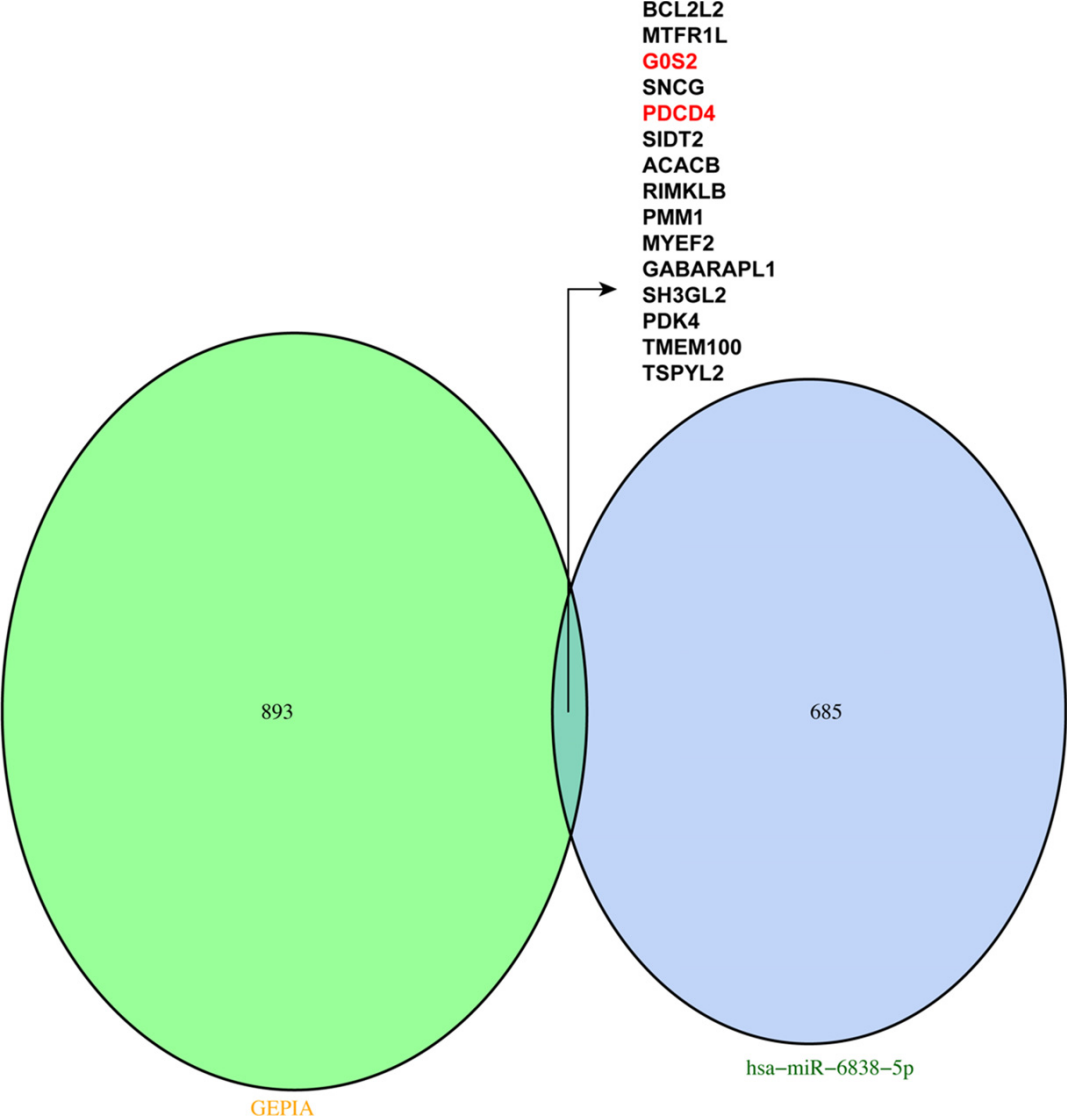
Venn diagram based on 700 target genes and 908 differential genes from GEPIA.

**Fig. (9):**
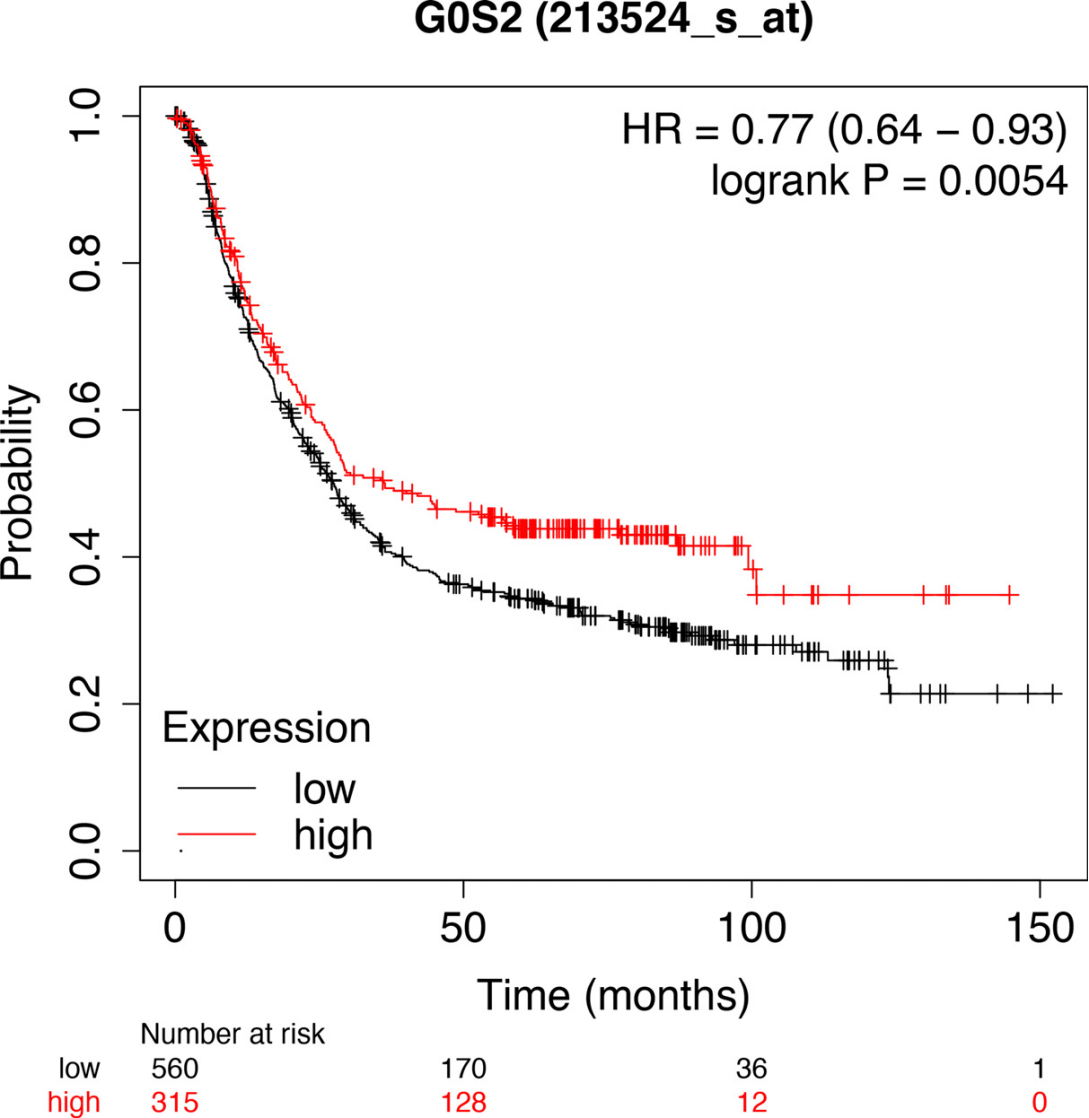
Kaplan-Meier curves for survival analyses of PDCD4 and G0S2. Produced by Kaplan Meier-plotter database.

**Fig. (10):**
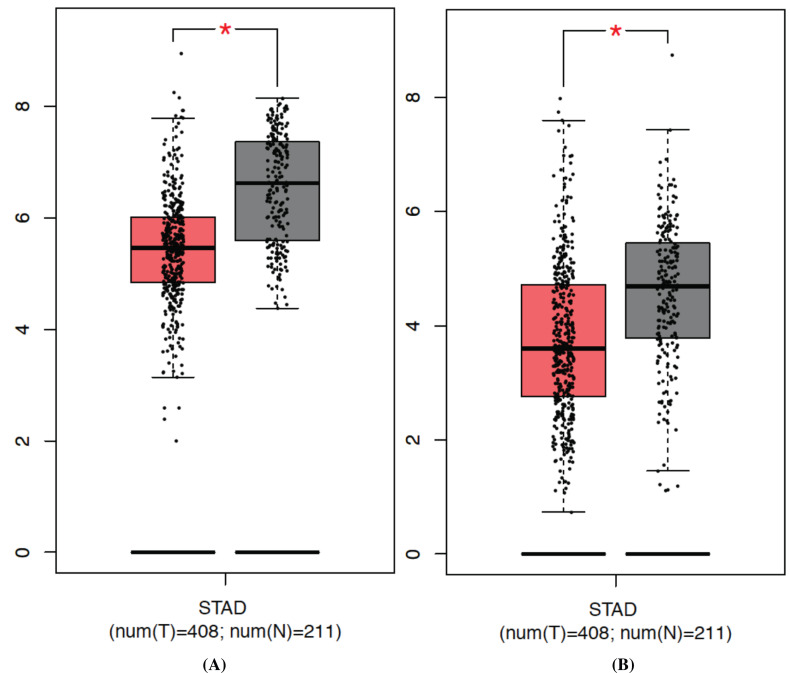
Relative expression of PDCD4 and G0S2 compared with gastric tumor and peritumoral tissue. (**A**), PDCD4; (**B**), G0S2.

**Fig. (11):**
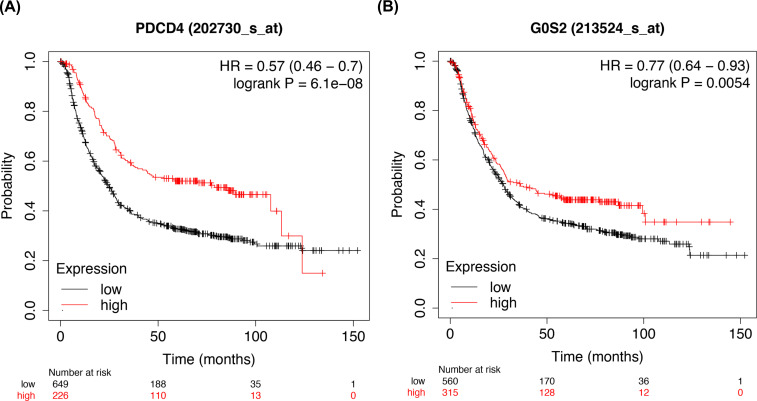
The different expression of PDCD4 and G0S2. (**A**), PDCD4; (**B**), G0S2. P roduced by GEPIA.

Based on the outcomes of the GEPIA database, there are 908 down-regulated genes related to GC. Combined the above 700 genes, we identified 15 target genes that have tightly association with hsa-miR-6838-5p (Fig. **8**). Then, we found that G0S2 and PDCD4 were downregulated in the tumor tissue by performing qRT-PCR (Figs. **9** and **10**). Thus, G0S2 and PDCD4 were identified as the two potential target genes. Further investigation indicated that the survival rates were dramatically lower in G0S2 and PDCD4 low expression groups compared to the high expression groups with *P* value less than 0.05 (Fig. **11**).

## 4 DISCUSSION

Gastric cancer is still the second leading cause of cancer-related mortality in China because of its highly malignant nature [27]. The understanding of the molecular pathogenesis of gastric cancer has advanced considerably in the last decades. With the recent development of transcriptome sequencing technology, circRNAs have attracted extensive attention from researchers. Mounting pieces of evidence confirmed that circRNAs participate in various bioprocesses in many tumors, such as hepatocellular carcinoma, Breast cancer, non-small-cell lung cancer [28], and bladder cancer [29]. However, few circRNAs have been well mechanistically featured in gastric cancer, and the biological functions of most circRNAs have yet to be elucidated. In our study, we identified a novel circRNA circSNTB2, which is dramatically down-regulated in gastric cancer tissues by analyzing the microarray from gastric cancer tissues and adjacent normal tissues. The under-expression of circSNTB2 in gastric cancer indicates that gastric cancer may be inhibited by up-regulating circSNTB2.

CircRNAs have been proven to be the competitive endogenous RNAs (ceRNAs) that could target miRNAs by acting as a miRNA sponge to be involved in human diseases [30]. Here, we predicted that miR-6938-5p as the target of circSNTB2 by analyzing the online database. Furthermore, the expression of miR-6938-5p was found to be elevated in gastric cancer tissue compared with the normal tissue. Gene function analysis, including GO analysis and KEGG pathway analysis was performed for the target mRNAs of the miR-6938-5p. The results of KEGG pathway analysis suggested that PI3K-Akt signaling pathway, MAPK signaling pathway, Human papillomavirus infection, mTOR signaling pathway were significantly enriched which have been already known as the key signals in regulating several cancers including gastric cancer [19, 31-33].

Further mechanism exploration revealed that G0S2 and PDCD4 act as the target genes of miR-6938-5p. G0S2 was discovered in the early 1990s by Russell and Forsdyke in cultured mononuclear cells which only exist in vertebrates [34]. Many studies have uncovered that G0S2 is a multifaceted protein implicated in cell proliferation, apoptosis, metabolism, and carcinogenesis [35]. Yim has reported that low G0S2 expression in breast cancer, particularly estrogen receptor-positive breast cancer, correlates with increased rates of its recurrence [36]. Besides, G0S2 could function as a tumor suppressor by opposing c-Myc [37]. PDCD4 is another novel tumor suppressor with multi-functions inhibiting tumor cell proliferation, tumor invasion and metastasis [38]. Decreased expression of PDCD4 has been strongly believed to be implicated in the development and progression of many kinds of human tumors, including lung, colon, liver, and breast cancer [39-42]. Our findings highlighted that the expression of both G0S2 and PDCD4 were inhibited in gastric cancer tissues while compared with the normal tissues. Consistently, the survival rates in the decreased expression of G0S2 and PDCD4 group were significantly lower than G0S2 and PDCD4 high expression group. These results depicted that G0S2 and PDCD4 function as the aim genes of miR-6938-5p playing a central role in the development and prognosis of GC.

## CONCLUSION

Taken together, the present data illustrated that circRNAs are aberrantly expressed in GC. Significant down-regulation of hsa_circ_0040039 (circSNTB2) expression in GC tissues was confirmed. Mechanism exploration suggested circSNTB2 can serve as the ceRNA to sponge miR-6938-5p to participate in GC tumorigenesis by modulating the expression of G0S2 and PDCD4. Our research characterized the regulation of circSNTB2/miR-6938-5p/G0S2 and PDCD4 pathways and their role in gastric cancer. In the future, a longitudinal study is needed to perform to identify the potential of circSNTB2 as a biomarker for GC diagnosis and targeted therapy.

## AUTHORS’ CONTRIBUTIONS

Yongkun Zhou and Qingying Sun designed the study. Baohai Rong wrote the manuscript. Xiqi Chen, Guangdong Xie and Letian Han collected all data. Hanhan Chen and Baohai Rong analyzed the collected data.

## Figures and Tables

**Table 1:** Primer sequences.

Gene	Sequence
PDCD4-F	ACTGTGCCAACCAGTCCAAAGG
PDCD4-R	CCTCCACATCATACACCTGTCC
G0S2-F	GCCTGATGGAGACTGTGTGCAG
G0S2-R	TCCTGCTGCTTGCCTTTCTCCT

**Table 2:** Top 10 of upregulated circRNAs.

Circbase ID	logFC	AveExpr	*P*-value	FDR	Expression
hsa_circ_0008035	2.91927821	6.89227094	0.00058292	0.02451527	up
hsa_circ_0023642	2.70028054	7.41701283	0.00013856	0.01182605	up
hsa_circ_0000144	2.6228974	7.50028727	3.38E-05	0.00629252	up
hsa_circ_0061274	2.56455241	7.50872483	0.0002009	0.01384334	up
hsa_circ_0032821	2.563427	6.60175864	0.00121676	0.0343536	up
hsa_circ_0005529	2.4402455	6.42675127	4.15E-05	0.00633536	up
hsa_circ_0084171	1.96803569	6.39667979	0.00345075	0.0515035	up
hsa_circ_0000098	1.61044589	11.91500096	0.00497559	0.05869578	up
hsa_circ_0004099	1.59791664	11.3655621	0.00198951	0.04094725	up
hsa_circ_0092306	1.39160562	9.29371962	6.20E-05	0.00746321	up

**Table 3:** Top 10 of downregulated circRNAs.

Circbase ID	logFC	AveExpr	*P*-value	FDR	Expression
hsa_circ_0040039	-3.4304697	8.33704363	2.63E-06	0.00154393	down
hsa_circ_0068610	-3.0174745	8.1299244	3.64E-05	0.00629252	down
hsa_circ_0000026	-2.9291308	7.40024291	4.11E-05	0.00633536	down
hsa_circ_0041732	-2.8408163	8.84728197	8.97E-05	0.00839862	down
hsa_circ_0005927	-2.8228718	8.7355341	1.88E-06	0.00154393	down
hsa_circ_0092341	-2.7346717	8.12546551	3.70E-06	0.00178928	down
hsa_circ_0001561	-2.6786923	9.72561231	0.0006933	0.02514962	down
hsa_circ_0092342	-2.4794679	7.96737911	2.09E-05	0.00552248	down
hsa_circ_0009172	-2.4711834	9.10137807	3.73E-07	0.00108171	down
hsa_circ_0067871	-2.4561799	7.87561576	0.00066535	0.02474272	down

## Data Availability

Tha authors confirm that the data and supportive information are available within the article.

## LIST OF ABBREVIATIONS

circRNAs: Circular RNAs
GO: Gene Ontology
BP: Biological Process
MF: Molecular Function
CC: Component Cellular
KEGG: Kyoto Encyclopedia of Genes and Genomes pathways
STAD: Stomach Adenocarcinoma
